# Student tutors for hands-on training in focused emergency echocardiography – a randomized controlled trial

**DOI:** 10.1186/1472-6920-12-101

**Published:** 2012-10-29

**Authors:** Matthias Kühl, Robert Wagner, Markus Bauder, Yelena Fenik, Reimer Riessen, Maria Lammerding-Köppel, Meinrad Gawaz, Suzanne Fateh-Moghadam, Peter Weyrich, Nora Celebi

**Affiliations:** 1 University Hospital Tübingen, Department of Internal Medicine, Division of Cardiology, University Hospital of Tübingen, Otfried-Müller-Str. 10, 72076, Tübingen, Germany; 2 University Hospital Tübingen, Department of Internal Medicine, Division of Diabetes, Endocrinology, Angiology, Nephrology and Clinical Chemistry, University Hospital of Tübingen, Otfried-Müller-Str. 10, 72076, Tübingen, Germany; 3 University of Tübingen, Medical School, Geisweg 3, 72076, Tübingen, Germany; 4 University Hospital Tübingen, Medical Intensive Care Unit, University Hospital of Tübingen, Otfried-Müller-Str. 10, 72076, Tübingen, Germany; 5 Competence Center for Medical Didactics, Elfriede-Aulhorn-Straße 10, 72076, Tübingen, Germany

## Abstract

**Background:**

Focused emergency echocardiography performed by non-cardiologists has been shown to be feasible and effective in emergency situations. During resuscitation a short focused emergency echocardiography has been shown to narrow down potential differential diagnoses and to improve patient survival. Quite a large proportion of physicians are eligible to learn focused emergency echocardiography. Training in focused emergency echocardiography usually comprises a lecture, hands-on trainings in very small groups, and a practice phase. There is a shortage of experienced echocardiographers who can supervise the second step, the hands-on training. We thus investigated whether student tutors can perform the hands-on training for focused emergency echocardiography.

**Methods:**

A total of 30 volunteer 4th and 5th year students were randomly assigned to a twelve-hour basic echocardiography course comprising a lecture followed by a hands-on training in small groups taught either by an expert cardiographer (EC) or by a student tutor (ST). Using a pre-post-design, the students were evaluated by an OSCE. The students had to generate two still frames with the apical five-chamber view and the parasternal long axis in five minutes and to correctly mark twelve anatomical cardiac structures. Two blinded expert cardiographers rated the students’ performance using a standardized checklist. Students could achieve a maximum of 25 points.

**Results:**

Both groups showed significant improvement after the training (p < .0001). In the group taught by EC the average increased from 2.3±3.4 to 17.1±3.0 points, and in the group taught by ST from 2.7±3.0 to 13.9±2.7 points. The difference in improvement between the groups was also significant (p = .03).

**Conclusions:**

Hands-on training by student tutors led to a significant gain in echocardiography skills, although inferior to teaching by an expert cardiographer.

## Background

Focused emergency echocardiography performed by non-cardiologists has been shown to be feasible and useful in emergency situations [1-3]. Aichinger et al. investigated the effect of a focused emergency echocardiography by sonography-inexperienced physicians in a prehospital setting. The acquisition of diagnostic images was possible in all 42 cases. With focused emergency echocardiography it was possible to distinguish between the patients with or without cardiac movement. Patients with cardiac movements survived until hospital admission in 40%, while only 3% of patients with cardiac standstill survived [4]. In a prospective study by Breitkreutz et al., sonography-inexperienced emergency physicians performed a focused emergency echocardiography on 230 patients during resuscitation or in shock state. Images of diagnostic quality were obtained in 94%. In 78% the additional information was derived from echocardiography altered management [5]. In a study by Prosen et al., focused emergency echocardiography in combination with end-tidal capnography during resuscitation of patients with pulseless electrical activity guided the vasopressor therapy. This led to an improved outcome and restoration of spontaneous circulation in 94% of the patients in comparison to historical controls (54%). Even the rate of a good neurological outcome among the survivors was significantly better (50% versus 8%) [6]. There are anecdotal reports about focused emergency echocardiography helping to identify pericardial tamponade [7].

Even in non-resuscitation situations, focused emergency echocardiography has been shown to be useful, for example to distinguish sepsis induced myocardial dysfunction from volume depletion in patients with septic shock and thus guiding fluid therapy [8]. The value of focused emergency echocardiography has been especially shown for paediatric emergencies [9,10].

In consequence, quite many physicians are candidates to learn focused emergency echocardiography. The skill might be useful for example for emergency physicians, intensivists, paediatricians, surgeons, anaesthesiologists and non-cardiologic internists even in early stages of their career. Point of care ultrasound is receiving increasingly more attention and its use for various indications as a supplementation to physical examination is spreading fast [11]. Therefore basic ultrasound techniques like focused emergency echocardiography should probably be taught in medical school along with ECG interpretation and physical examination. The desired level of competence is the one below level 1 as proposed by Price et al., that is the acquisition of the standard transthoracic echocardiographic views, the recognition of the major causes for cardiac arrest and shock and recognition when referral for a second opinion is indicated [12].

Several training modalities for focused emergency echocardiography (for example FEEL: focused echocardiographic evaluation in life support and FATE: focused assessment with transthoracic echocardiography) have been proposed, lasting two hours to one day [4,12,13]. Hofer et al. propose a lecture for a large group followed by hands on-trainings in very small groups as the desired teaching technique [14]. The learning goal of the hands on-training is the ability to obtain diagnostic images of the standard views in which the anatomical landmarks are clearly identifiable. The main obstacle in implementing focused emergency echocardiography into the medical school curricula is the shortage of experienced echocardiographers who can supervise the small group hands-on trainings.

Peer-assisted learning has been shown to be an appropriate teaching concept for small group tutorials in various contexts such as problem oriented learning, technical skills, and musculoskeletal and abdominal ultrasound [15-19]. However, echocardiography is a rather complex and difficult skill to both teach and to learn. As the functional and dynamic aspects of echocardiography are much more complicated than the aforementioned skills, we cannot assume that such a peer assisted learning model is adequate without further research.

We thus investigated whether student tutors can effectively teach the hands-on part of focused emergency echocardiography to medical students without prior echocardiographic training compared to expert echocardiographers using a prospective, randomized, controlled study design.

## Methods

### Study design and participants

A total of 30 volunteer medical students (3rd-5th year) without prior echocardiographical training were randomized into two equally large groups using a table with random numbers. One group was taught by student tutors (ST), the other by expert cardiographers (EC). The students were blinded to the study question. Both ST and EC introduced themselves with their first names only and oversaw the hands-on-training without explaining their qualification.

Before and after the course the students’ echocardiographical abilities were assessed with the same test (see next section).

### Echocardiography course

The echocardiography course comprised three 45-min lectures in a large group, introducing the basics of echocardiography, the standard sections and the anatomical landmarks followed by three 135-min hands-on training sessions, in which students practiced echocardiography on each other. The student/instructor (ST or EC) ratio was 1:3 in each group.

In the echocardiography course EC and ST followed a predefined course program comprising the following learning goals:

1. Lectures:

a. Fundamentals of 2-D and Doppler ultrasound (Doppler shift, aliasing, etc).

b. Recognition of structures in different image sections.

c. Normal range of important cardiac parameters (systolic and diastolic diameters, pressure gradients over atrioventricular valves, etc.).

2. Hands on-trainings:

a. Correct positioning and displaying of the most important echocardiographic ultrasound image sections (parasternal short axis, parasternal long axis, four- and five-chamber views from the apical acoustic window, and finally the subcostal acoustic window).

b. Important measurements: left ventricular M-Mode, measurements of the aortic root and the left atrium; measurements of the LVEDD and RVEDD (including septum thickness) in a 2-D still frame; color Doppler of all 4 valves; diameter measurement of the inferior vena cava (IVC).

The desired level of competence was to be able to generate the standard views, to recognize anatomical structures and major pathological findings such as pericardial tamponade, a severely reduced ejection fraction or overt valve disease. More advanced skills such as grading the valve disease were not part of the training.

After the course the students rated the overall satisfaction with the course and their teachers on a six-point Likert scale (1 = very good, 6 = not sufficient).

### Assessment

We assessed the students with a five-minute OSCE (objective structured clinical exam) before and after the course. The expert cardiographers and the student tutors optimized the settings of the echocardiography machine (ACUSON X 300 PE, Version 7.0, Siemens Healthcare, Erlangen, Germany), and chose the leanest students in the groups as subjects for the echocardiographical examination. The students were asked to produce a still-frame of the apical five-chamber view and the parasternal long axis and to label the structures they recognized. Points were given for every one of the twelve structures that was recognizable and correctly labeled in the five-chamber section (maximum: twelve points), the correct depiction of the anterior and posterior mitral valve cusp and regurgitation jet of the mitral valve (maximum: three points) and overall quality of the images (five point per image, maximum: ten points). The images were rated by two blinded expert cardiographers using a checklist.

### Student tutors

Six student tutors (3rd to 6th year) received an echocardiography training comprising of:

• A five hour introductory seminar with theoretical background on echocardiography and practical exercises simulating the standard echocardiographical examination.

• A three-week fulltime practical (eight hours per day) with expert echocardiographers.

• A three-hour meeting with an expert echocardiographer to demonstrate the echocardiographical expertise and to simulate part of the course.

• A twelve-hour didactic seminar [20] standardized for the peer-assisted learning model implemented at our faculty.

### Ethical issues

The study protocol was reviewed and accepted by the local ethics committee. Students were only chosen to serve as subjects for the echocardiographic exam if they had given written consent to be informed in case of a pathological finding. The images generated in the OSCE were encoded to ensure anonymity. Study participation was voluntary.

### Statistics

The OSCE-scores of the pre-test had a skewed distribution. All other examined parameters had normal distribution in the Shapiro-Wilk test.

The knowledge-gain of the ST and EC was compared using a parametrical t-test. The “two one-sided t-tests” (TOST) method was used as a test of equivalence [21]. Results are shown as mean ± standard deviation. A difference of less than 10% (2.5 points) was considered irrelevant. The interrater reliability was calculated as interclass correlations and the corresponding 95%-confidence intervals according to Shrout and Fleiss [22].

## Results

Students’ characteristics are shown in Table 1.

**Table 1:** Characteristics of the study participants, none of the students had systematic echocardiography training before

	**Students taught by expert echocardiographers**	**Students taught by student tutors**
Age	23.9 ± 1 years	24.9 ± 2 years
Gender	5 m, 10 f	4 m, 11 f
Year	7 4th year	3 3rd year
	8 5th year	6 4th year
		6 5th year
Echocardiography observed	11 < 3 times	12 < 3 times
	3 > 3 times	0 > 3 times
	1 > 10 times	3 > 10 times
Echocardiography performed	12 never	13 never
	0 once	1 once
	3 > 3 times	1 > 3 times

Students’ characteristics.

In the post-hoc power analysis for independent groups, the effect size was .83, with n = 30 and α = .05. The power to detect a difference between the groups was .72.

The interrater-reliability of the two blinded expert echocardiographers rating the OSCE images was .96 (0.941 – 0.979).

**Figure 1:**
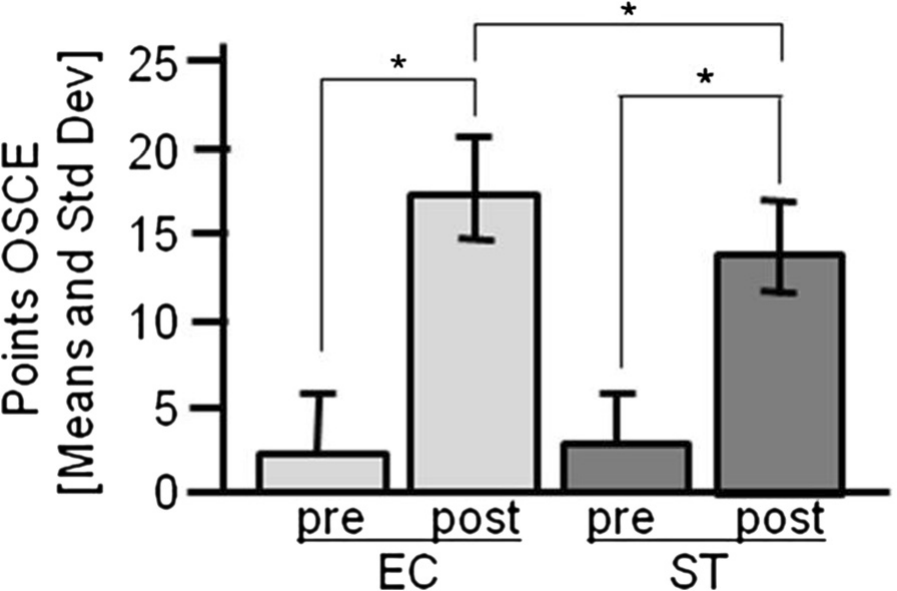
**OSCE Scores.** OSCE-Scores of the medical students before and after the hands-on training supervised by expert echocardiographers (EC) and student tutors (ST).

Both groups improved significantly after the training (p < .0001). In the group taught by EC, the average OSCE scores increased from 2.3±3.4 to 17.1±3.0 points, and in the group taught by ST from 2.7±3.0 to 13.9±2.7 points (see Figure 1). The difference in improvement between the groups was also significant (p = .03).

The EC and ST were rated on a six point Likert scale (1 = very good, 6 = not sufficient) by their students. The EC received 1.3±.4 points and the ST 1.4±.5 points.

## Discussion

In our study we compared the skill gain in obtaining correct still frames as used in focused emergency echocardiographic exam by hands-on trainings supervised by student tutors versus expert echocardiographers. In both groups there was a increase in skill, but the group that was taught by the expert echocardiographers scored significantly better. Thus, hands on-training supervised by student tutors is probably inferior to hands on-training by expert echocardiographers for focused emergency echocardiography skills.

In previously published studies comparing the peer assisted learning concept with faculty teaching there was no difference in knowledge and skill gain, even when using ultrasound techniques [15,16,23,24]. This finding may be due to the complexity of echocardiography in comparison to the aforementioned skills. The student tutor training was probably too short, or the student tutors needed more time to teach the same skills compared to expert echocardiographers. Another possibility is that the contents of our echocardiography training were too ambitious. In addition to the FATE and FEEL concepts, we also taught the students to recognize overt valve disease (without grading). In a study by Alexander et al. from the Duke University novice echocardiographers with a portable ultrasound device had a good agreement to gold standard echocardiography after a three hour training for major findings (i.e. pericardial effusion, aortic valve immobility), but only a moderate agreement for less overt pathologies (i.e. moderate or severe left ventricular dysfunction, mitral valve regurgitation) [25].

Since it requires a lot of practice to find the correct acoustic window for echocardiography, the student tutors probably took longer to demonstrate the correct position and to correct the students, thus limiting the students’ practice time. It is not clear, whether this can be counterbalanced with a more extensive training for the student tutors.

This raises the question as to which degree of complexity can be sufficiently covered by peer assisted learning involving student tutors who generally lack a broader clinical experience. At our faculty, aside from this course on echocardiography, abdominal sonography and central line catheterization are the most complex procedural skills taught by student tutors on a simulator at the skills lab. We consider peer assisted learning to be sufficient to teach the basics of these advanced skills. However, it has yet to be determined which degree of complexity in procedural skills can or cannot be taught by peer assisted learning. On the other hand, procedural skills lab training only prepares students for their upcoming clinical activities. It cannot be a substitute for expert knowledge arising from years of clinical practice and it is designed to prepare for practice, not to substitute practice. Therefore, it is of doubt whether the rather small difference in knowledge gain between EC and ST in this study is really relevant for future clinical activities of the medical students.

Our study has several limitations: first of all, it is a single centre experience with a small sample size. The OSCE comprised only parts of the whole focused emergency echocardiographic exam and the allotted time was limited in order to standardize the examination conditions. Our aim was to assess the ability to find the correct acoustic window and to recognize the anatomical cardiac structures, so the assessment did not comprise pathological findings. In addition, we assessed the echocardiography skills immediately after the lessons, so there are no data on long term retention.

Future research is needed to determine whether it is feasible to integrate focused emergency echocardiography into medical school curricula.

## Conclusions

Hands-on training supervised by student tutors led to a significant gain in echocardiography skills in echocardiography novices, although inferior to teaching by an expert cardiographer.

## Competing interests

The authors declare that they have no competing interests.

## Authors’ contributions

MK designed the study, taught the students as expert echocardiographer and drafted the manuscript. RW performed the statistics and revised the manuscript critically. MB taught the students as student tutor and contributed to data acquisition and study design. YF taught the students as student tutor and contributed to the manuscript including proof reading as native speaker. RR rated the still frames and revised the manuscript critically. PW rated the still frames, contributed to the study design and helped drafting the manuscript. MLK developed and performed the didactical training of the student tutors and revised the manuscript critically. MG instructed the student tutors in echocardiography and revised the manuscript critically. SFM instructed the student tutors in echocardiography and revised the manuscript critically. NC conceived of the study and helped drafting the manuscript. All authors critically read and approved of the final manuscript.

## Pre-publication history

The pre-publication history for this paper can be accessed here:

http://www.biomedcentral.com/1472-6920/12/101/prepub
